# Maternal High Fructose Intake Increases the Vulnerability to Post-Weaning High-Fat Diet-Induced Programmed Hypertension in Male Offspring

**DOI:** 10.3390/nu10010056

**Published:** 2018-01-09

**Authors:** You-Lin Tain, Wei-Chia Lee, Kay L. H. Wu, Steve Leu, Julie Y. H. Chan

**Affiliations:** 1 Department of Pediatrics, Kaohsiung Chang Gung Memorial Hospital and Chang Gung University College of Medicine, Kaohsiung 833, Taiwan; tainyl@hotmail.com; 2 Institute for Translational Research in Biomedicine, Kaohsiung Chang Gung Memorial Hospital and Chang Gung University College of Medicine, Kaohsiung 833, Taiwan; wlh0701@yahoo.com (K.L.H.W.); leu@mail.cgu.edu.tw (S.L.); 3 Department of Urology, Kaohsiung Chang Gung Memorial Hospital and Chang Gung University College of Medicine, Kaohsiung 833, Taiwan; dinor666@ms32.hinet.net

**Keywords:** developmental origins of health and disease (DOHaD), high-fat, fructose, hypertension, nutrient sensing signal, oxidative stress, soluble epoxide hydrolase

## Abstract

Widespread consumption of high-fructose and high-fat diets relates to the global epidemic of hypertension. Hypertension may originate from early life by a combination of prenatal and postnatal nutritional insults. We examined whether maternal high-fructose diet increases vulnerability to post-weaning high-fructose or high-fat diets induced hypertension in adult offspring and determined the underlying mechanisms. Pregnant Sprague-Dawley rats received regular chow (ND) or chow supplemented with 60% fructose (HFR) during the entire pregnancy and lactation periods. Male offspring were onto either the regular chow, 60% fructose, or high-fat diet (HFA) from weaning to 12 weeks of age and assigned to four groups: ND/ND, HFR/ND, HFR/HFR, and HFR/HFA. Maternal high-fructose diet exacerbates post-weaning high-fat diet-induced programmed hypertension. Post-weaning high-fructose and high-fat diets similarly reduced *Sirt4*, *Prkaa2*, *Prkag2*, *Ppara*, *Pparb*, and *Ppargc1a* mRNA expression in offspring kidneys exposed to maternal high-fructose intake. Additionally, post-weaning high-fat diet significantly reduced renal mRNA levels of *Ulk1*, *Atg5*, and *Nrf2* and induced greater oxidative stress than did high-fructose diet. Although maternal high-fructose intake increases soluble epoxide hydrolase (SEH) expression in the kidney, which was restored by post-weaning high-fructose and high-fat diets. Maternal high-fructose diet programs differential vulnerability to developing hypertension in male offspring in response to post-weaning high-fructose and high-fat diets. Our data implicated that specific therapy targeting on nutrient sensing signals, oxidative stress, and SEH may be a promising approach to prevent hypertension in children and mothers exposed to high-fructose and high-fat consumption.

## 1. Introduction

Over consumption of the Western diet, characterized by high refined sugars and fat content, is a major contributor to the global epidemic of hypertension. Fructose is a monosaccharide naturally present in fruits and vegetables. Yet modern-day humans have an almost insatiable appetite for fructose coming from refined sugars and processed foods. The worldwide per capita fructose consumption has grown in the last century and its growth has been paralleled by an increase in hypertension [1].

Hypertension can be driven by early-life adverse conditions by the so-called developmental origins of health and disease (DOHaD) [2]. Hypertension is a multifactorial disorder and blood pressure (BP) is governed primarily by the kidney. While the developing kidney is vulnerable, in particular, to adverse environments in early life, initiating permanent structural and physiological adaptions, namely, renal programming [3]. Our previous reports showed that offspring rats of mothers exposed to 60% high-fructose diet during pregnancy and lactation developed renal programming and hypertension [4,5], which is in agreement with the results of earlier studies involving fructose-fed adult rats [6].

On the other hand, high-fat diets have been a focus of investigation in the area of hypertension [7]. Postnatally nutritional insult can act as a second hit to intensify programmed outcomes of a first hit [8]. We previously found that post-weaning high-fat diet exacerbates prenatal dexamethasone exposure-induced programmed hypertension in adult offspring [9]. Nevertheless, whether postnatal high-fructose or high-fat diets can enhance vulnerability to maternal high-fructose intake-induced programmed hypertension in adult offspring remains unknown.

Maternal high-fructose intake-induced renal programming and hypertension have been attributed to some mechanisms, including nutrient sensing signals, oxidative stress, nitric oxide (NO) pathway, and arachidonic acid metabolites [4,10,11,12]. In response to nutrient intake, the nutrient sensing pathway maintains energy homeostasis and mediates autophagy [13]. Quite a few nutrient-sensing signals exist in the kidney, including SIRT (silent information regulator transcript), the peroxisome proliferator-activated receptor (PPAR) pathway, and adenosine monophosphate (AMP)-activated protein kinase (AMPK) pathway. On the other hand, arachidonic acid metabolites play an important role on the development of hypertension [14,15]. Arachidonic acid can be metabolized by CYP450 enzymes to produce epoxyeicosatrienoic acid (EETs). EETs are metabolized by soluble epoxide hydrolase (SEH) to generate dihydroxyeicosatrienoic acids (DHETs) in favor of vasoconstriction [15]. Our previous report showed that maternal high-fructose intake upregulates SEH in the adult offspring kidney and SEH inhibition protects against the development of hypertension in offspring rats of mothers exposed to high-fructose consumption [4,11]. In the present study we, therefore, investigated whether maternal high-fructose diet increases the vulnerability to post-weaning high-fructose, or high-fat diets induced hypertension in adult offspring via mediating nutrient sensing signals, oxidative stress, and SEH with a focus on the kidney. 

## 2. Materials and Methods 

### 2.1. Animal Model

This study was conducted in accordance with the Guideline for the Care and Use of Laboratory Animals by the US National Institutes of Health. All experimental protocols were approved by the Institutional Animal Care and Use Committee of Chang Gung Memorial Hospital, Kaohsiung. Virgin Sprague-Dawley (SD) rats were obtained from BioLASCO Taiwan Co., Ltd. (Taipei, Taiwan). Animals were housed in an Association for Assessment and Accreditation of Laboratory. Animal Care International (AAALAC)-approved animal facility in our hospital with controlled temperature and light cycle (12/12 light cycle). Male SD rats were caged with female rats until mating was confirmed by examining vaginal plugs.

Pregnant SD rats received regular chow (ND; *n* = 3) or chow supplemented with 60% fructose (HFR; *n* = 9) during the entire period of pregnancy and lactation [4]. In order to equal the received quantity of milk and maternal pup care, litters were standardized to eight pups per litter at birth. Only male offspring were selected from each litter and used in subsequent experiments because hypertension occurs at an earlier age and a higher rate in males than females [16]. Male offspring were assigned to four groups (maternal diet/post-weaning diet; *n* = 8 from three independent litters/group): ND/ND, HFR/ND, HFR/HFR, and HFR/high-fat diet (HFA). The offspring were weaned at three weeks of age, and onto either the regular chow (ND), 60% fructose (HFR), or high-fat diet (HFA; D12331, Research Diets, Inc., New Brunswick, NJ, USA; 58% fat (hydrogenated coconut oil) plus high sucrose (25% carbohydrate)) ad libitum from weaning to three months of age.

BP was measured in conscious and previously trained offspring at 3, 4, 6, 8, 10, and 12 weeks of age. BP was measured by the tail cuff method as detailed previously (BP-2000; Visitech Systems, Inc., Apex, NC, USA) [4]. To ensure accuracy and reproducibility, the rats were acclimated to restraint and tail-cuff inflation for one week before the experiment. For each rat, five measurements were recorded at each time point. Three stable consecutive measures were taken and averaged. The offspring rats were sacrificed at 12 weeks of age. Rats were anesthetized by intraperitoneally injecting ketamine (50 mg/kg body weight) and xylazine (10 mg/kg body weight) and were euthanized by intraperitoneally injecting an overdose of pentobarbital. Heparinized blood samples were collected. The kidneys were harvested after perfusion, divided into cortex and medulla, and stored at −80 °C for further analysis. Blood creatinine level was measured by high-performance liquid chromatography (HPLC).

### 2.2. Detection of l-Arginine, l-Citrulline, and Dimethylarginines by Performing HPLC

Plasma l-arginine, l-citrulline, asymmetric dimethylarginine (ADMA), and symmetric dimethylarginine (SDMA, a stereoisomer of ADMA) levels were determined by the HPLC (HP Series 1100; Agilent Technologies, Inc., Santa Clara, CA, USA) with o-phthaldialdehyde-3-mercaptopropionic acid derivatization reagent [4]. Concentrations of l-arginine, l-citrulline, ADMA, and SDMA in the standards were in the range of 1–100, 1–100, 0.5–5, and 0.5–5 μM, respectively.

### 2.3. Quantitative Real-Time Polymerase Chain Reaction

RNA was extracted from kidney cortex according to methods described previously [4]. Two-step quantitative real-time polymerase chain reaction (qPCR) was conducted using QuantiTect SYBR Green PCR Reagents (Qiagen, Valencia, CA, USA) on an iCycler iQ Multi-color Real-Time PCR Detection System (Bio-Rad, Hercules, CA, USA). We analyzed a number of genes involved in the nutrient-sensing pathway and autophagy, including sirtuin-1 (*Sirt1*), sirtuin-4 (*Sirt4*), peroxisome proliferator-activated receptor (PPAR)-α (*Ppara*), -β (*Pparb*), and -γ (*Pparg*), PPARγ coactivator 1-α (PGC-1α encodes for *Ppargc1a*), protein kinase, AMP-activated, subunit-α2 (*Prkaa2*), -β2 (*Prkab2*), and -γ2 (*Prkag2*), serine/threonine kinases, UNC-51-like kinase-1 (*Ulk1*), and autophagy related gene 5 (*Atg5*). Additionally, NF-E2-related factor-2 (*Nrf2*), a key regulator of antioxidants, was analyzed. One gene *Ephx2*, involved in arachidonic acid metabolism, was also analyzed. The 18S rRNA gene (*Rn18s*) was used as a reference. Primer sequences are provided in Table 1. All samples were run in duplicate. For the relative quantification of gene expression, the comparative threshold cycle (C_T_) method was employed. The averaged C_T_ was subtracted from the corresponding averaged Rn18s value for each sample, resulting in ΔC_T_. ΔΔC_T_ was achieved by subtracting the average control ΔC_T_ value from the average experimental ΔC_T_. The fold-change was established by calculating $2^{-\Delta \Delta C_{T}}$ for experimental vs. reference samples. 

### 2.4. Western Blotting

Western blot analysis was done as we have previously described [4]. A list of antibodies used for Western blotting are provided in Table 2. Bands of interest were visualized using enhanced chemilumescent reagents (PerkinElmer, Waltham, MA, USA) and quantified by densitometry (Quantity One Analysis software; Bio-Rad, Hercules, CA, USA), as integrated optical density (IOD) after subtraction of background. The IOD was factored for Ponceau red staining to correct for any variations in total protein loading. The protein abundance was represented as IOD/PonS.

### 2.5. Immunohistochemistry

Paraffin-embedded tissue sectioned at 3 μm thickness. Tissue slides were deparaffinized with xylene and rehydrated in a series of ethanol solutions with decreasing concentrations. Following blocking with immunoblock (BIOTnA Biotech., Kaohsiung, Taiwan), the sections were incubated with an anti-8-hydroxydeoxyguanosine (8-OHdG) antibody (1:100, JaICA, Shizuoka, Japan) at room temperature for 2 h. Immunoreactivity was revealed by use of the polymer-horseradish peroxidase (HRP) labelling kit (BIOTnA Biotech) and 3,3′-diaminobenzidine (DAB) as the chromogen. The sections were lightly counterstained with hematoxylin and preserved under cover glass. Identical staining omitting incubation with primary antibody was used as a negative control. Reagent incubation times and antibody dilutions were identical in all experiments. Quantitative analysis of 8-OHdG-positive cells per microscopic field (400×) in the renal sections was performed as we described previously [11]. For SEH, we used a rabbit anti-rat SEH antibody (1:100, overnight incubation; Santa Cruz Biotechnology, Santa Cruz, CA, USA) for detection of SEH. Identical staining omitting incubation with primary antibody was used as a negative control.

### 2.6. Statistical Analysis

Data were represented as mean ± standard error of the mean (SEM). The Shapiro-Wilk normality test was used to determine normally distributed data. Most parameters were compared across the groups via one-way ANOVA with Tukey’s post hoc test for multiple comparisons. BP was analyzed using two-way repeated-measures ANOVA and Tukey’s post hoc test. All the analyses were performed using Statistical Package for the Social Sciences (SPSS) version 15.0 (SPSS Inc., Chicago, IL, USA). A *p* value of < 0.05 was considered statistically significant.

## 3. Results

Litter sizes were not significantly altered by high-fructose exposure of the maternal rat (pups per litter: ND group = 14 ± 0.8; HFR group = 15.5 ± 0.8). The mortality rate of male pups was not different among the four groups. As shown in Table 3, the HFR/HFR group had lower body weights compared with the other three groups. The kidney weights were lower in the HFR/ND and HFR/HFA groups vs. ND/ND group. The kidney weight-to-body weight ratios were highest in the HFR/HFR group, indicating post-weaning HFR, but not HFA, increases the vulnerability to develop renal hypertrophy (expressed as an increased tissue weight to body weight ratio) in offspring exposed to maternal HFR diet. However, plasma creatinine level was not different among the four groups. 

**Figure 1:**
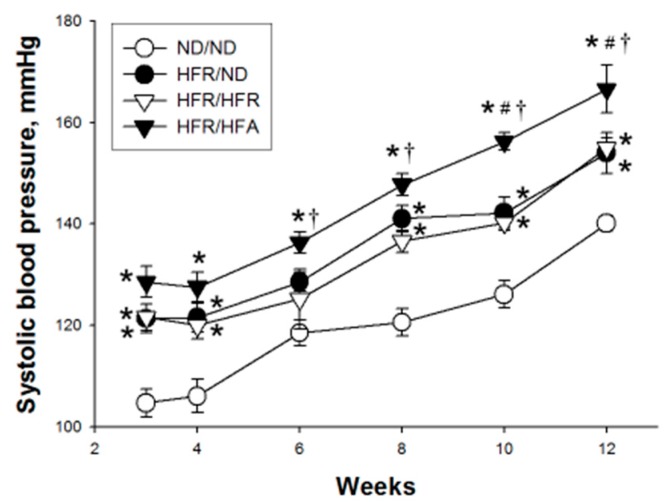
Effect of maternal and post-weaning high-fructose (HFR) and post-weaning high-fat (HFA) intake on systolic blood pressure in 12-week-old male offspring. * *p* < 0.05 vs. ND/ND; # *p* < 0.05 vs. HFR/ND; † *p* < 0.05 vs. HFR/HFR.

As shown in Figure 1, from three to 12 weeks of age, systolic BP significantly increased in HFR/ND, as well as in HFR/HFR offspring rats compared with that in ND/ND group and was the highest in HFR/HFA group. At 12 weeks of age, systolic and diastolic BPs, and mean arterial pressure, which were higher in both HFR/ND and HFR/HFR groups than those in ND/ND and HFR/ND group (Table 3). However, blood creatinine levels were not different among the four groups. These data indicated that post-weaning high-fat but not high-fructose aggravated maternal high-fructose intake induced programmed hypertension in 12-week-old offspring. However, maternal high-fructose, post-weaning high-fructose, and high-fat diets had no effect on renal function.

**Figure 2:**
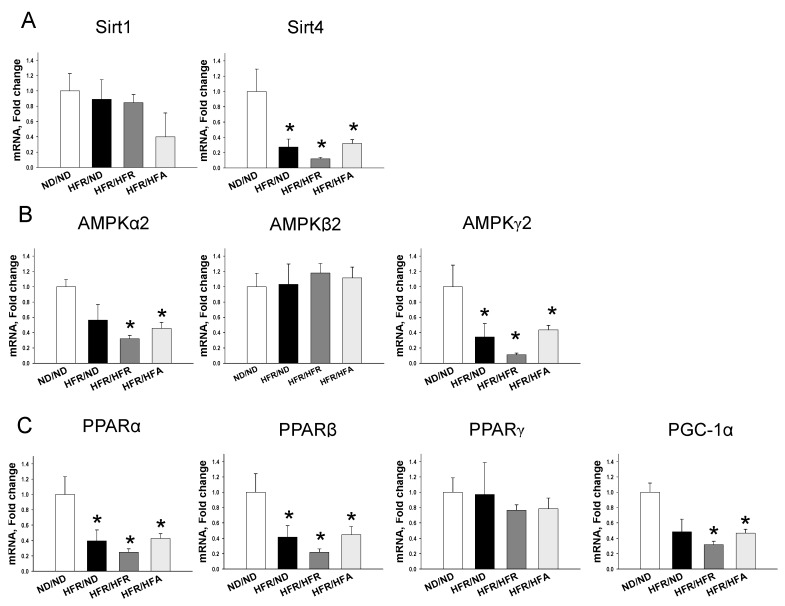
Effect of maternal and post-weaning high-fructose (HFR) and post-weaning high-fat (HFA) intake on mRNA expression of (**A**) silent information regulator transcript 1 (SIRT1) and 4 (SIRT4); (**B**) AMP-activated protein kinase (AMPK) α-, β-, and γ-subunits; and (**C**) peroxisome proliferator-activated receptor (PPAR) α-, β-, and γ-isoforms and PPARγ coactivator-1α (PGC-1α) in male offspring kidneys at 12 weeks of age. *n* = 8/group. * *p* < 0.05 vs. ND/ND.

**Figure 3:**
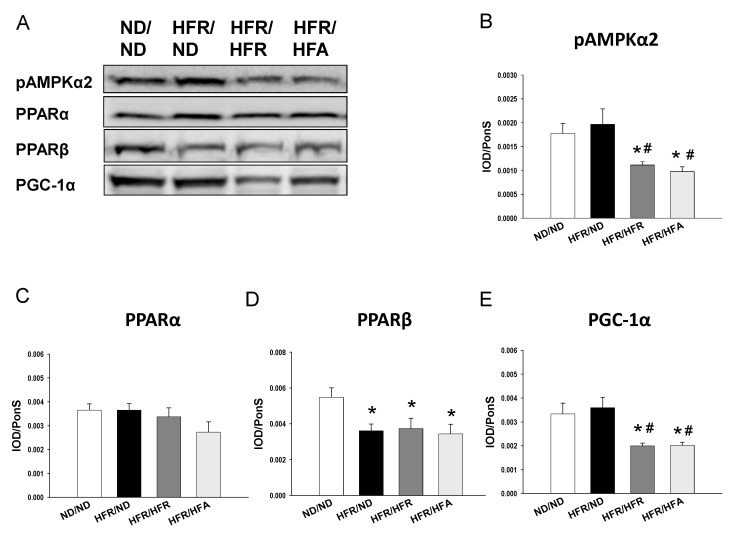
(**A**) Representative Western blots and relative abundance of (**B**) phosphor-AMPKα2 (63 kDa); (**C**) PPARα (52 kDa); (**D**) PPARβ (52 kDa); and (**E**) PGC-1α (90 kDa) in offspring kidneys at 12 weeks of age. *n* = 8/group. * *p* < 0.05 versus ND/ND; # *p* < 0.05 versus HFR/ND.

We first analyzed genes involved in the nutrient sensing pathway. As shown in Figure 2, renal mRNA expression of *Sirt4*, *Prkag2* (encoding for AMPKγ2), *Ppara* (encoding for PPARα), and *Pparb* (encoding for PPARβ) were higher in the ND/ND group compared to the other three groups. HFR/HFR and HFR/HFA similarly decreased mRNA expression of *Prkaa2* (encoding for AMPKα2) and *Ppargc1a* (encoding for PGC-1α) in offspring kidneys vs. the ND/ND group. Consistent with the change in mRNA level, renal protein level of phosphorylated AMPKα2 (Figure 3B) and PGC-1α (Figure 3E) were lower in the HFR/HFR and HFR/HFA group than those in the ND/ND group. Likewise, the protein level of PPARβ was higher in the ND/ND group compared to the other three groups. 

**Figure 4:**
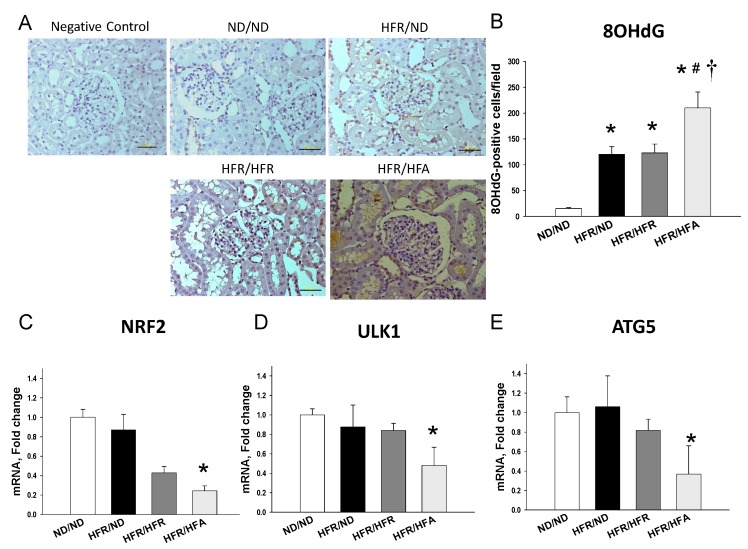
(**A**) Light micrographs illustrating immunostaining for 8-hydroxydeoxyguanosine (8-OHdG) in the kidney in male offspring at 12 weeks of age. Bar = 50 μm; (**B**) quantitative analysis of 8-OHdG-positive cells per microscopic field (400×); Effect of maternal and post-weaning high-fructose (HFR) and post-weaning high-fat (HFA) intake on mRNA expression of (**C**) *Nfr2*, (**D**) *Ulk1*, and (**E**) *Atg5* in male offspring kidneys at 12 weeks of age. *n* = 8/group. * *p* < 0.05 vs. ND/ND; # *p* < 0.05 vs. HFR/ND; † *p* < 0.05 vs. HFR/HFR.

As dysfunction of autophagy may result in abnormal mitochondrial function and oxidative stress, we next examined whether oxidative stress and autophagy are involved in high-fructose and high-fat-induced programmed hypertension. We evaluated oxidative stress in offspring kidneys by immunohistochemistry for 8-OHdG, a marker of oxidative DNA damage. As shown in Figure 4A,B, immunostaining of both cytoplasmic and nuclear 8-OHdG in the glomeruli and renal tubules indicated intense staining in the HFR/HFA group (210 ± 31 positive cells), an intermediate level of staining in the HFR/ND (120 ± 15 positive cells), as well as in the HFR/HFR (123 ± 17 positive cells) groups, and little staining in the ND/ND group (15 ± 2 positive cells). We observed that the combination of maternal high-fructose and post-weaning high-fat diets significantly decreased mRNA expression of *Nrf2* (Figure 4C), *Ulk1* (Figure 4D), and *Atg5* (Figure 4E) compared to the ND/ND group. These results suggest that increased oxidative stress and reduced autophagy are mainly attributed to maternal high-fructose plus post-weaning high-fat diets. 

The link between the ADMA-NO pathway and oxidative stress in programmed hypertension has been well studied [17]. Hence, we investigated whether high-fructose and high-fat diets induced an imbalance in the ADMA-NO pathway (Table 4). We observed that plasma l-citrulline, ADMA, and SDMA levels were not different among the four groups. However, post-weaning high-fructose and high-fat diets both caused the decreases of plasma l-arginine levels and l-arginine-to-ADMA ratios in offspring exposed to maternal high-fructose intake.

**Figure 5:**
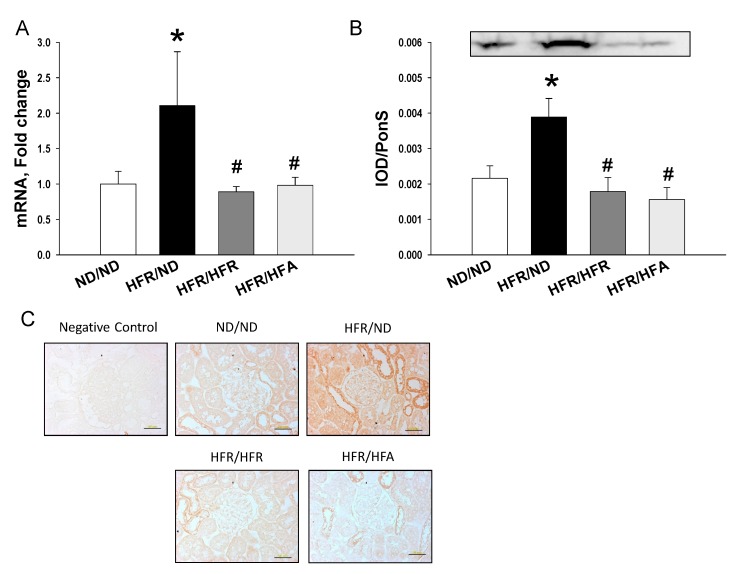
Effect of maternal and post-weaning high-fructose (HFR) and post-weaning high-fat (HFA) intake on mRNA expression of (**A**) *Ephx2* and (**B**) soluble epoxide hydrolase (SEH) protein (62 kDa); and (**C**) light micrographs illustrating immunostaining for SEH in the kidney in male offspring at 12 weeks of age. Bar = 50 μm. *n* = 8/group; * *p* < 0.05 vs. ND/ND; # *p* < 0.05 vs. HFR/ND.

Furthermore, we examined the alterations of SEH in response to post-weaning high-fructose and high-fat diets, as we previously found that SEH was involved in maternal high-fructose induced programmed hypertension [4,11]. Our data showed that renal *Ephx2* (encoding for SEH) mRNA expression (Figure 5A) and SEH protein level (Figure 5B) were higher in the HFR/ND group vs. ND/ND, while their levels were restored by post-weaning high-fructose or high-fat diet. Similarly, immunostaining of SEH in the glomeruli and tubules of offspring kidneys indicated intense staining in the HFR/ND group, while there was little staining in the HFR/HFR and the HFR/HFA groups. 

## 4. Discussion

This study provides insight into similar and differential mechanisms by which post-weaning high-fructose and high-fat diets induces renal programming and hypertension in adult offspring exposed to maternal high-fructose consumption. The major findings of our study can be summarized as follows: (1) post-weaning high-fat diet aggravates maternal high-fructose diet-induced programmed hypertension in 12-week-old male offspring; (2) post-weaning high-fructose did not either intensify or lessen maternal high-fructose induced programmed hypertension; (3) post-weaning high-fructose and high-fat diet-induced hypertension is related to nutrient-sensing signals, autophagy, oxidative stress, and NO pathway; (4) combined post-weaning high-fat diet and maternal high-fructose significantly reduced mRNA expression of *Nrf2*, *Ulk1*, and *Atg5* in offspring kidneys; and (5) maternal high-fructose intake increases SEH expression in the kidney, which is restored by post-weaning high-fructose and high-fat intake.

We found that there is a synergistic effect between maternal high-fructose diet and post-weaning high-fat diet causing a rise on BP in support of our previous study showing that maternal high-fructose intake can amplify adverse effects of postnatal insult on BPs in adult offspring [18]. However, the combination of maternal and post-weaning high-fructose diets brought about a negligible synergistic effect on BPs. The present study is consistent with previous reports showing that prenatal insult did not either intensify or lessen post-weaning insult induced programmed hypertension if both insults are the same [19,20]. Our data imply that the effect of maternal nutritional insults on the fetus are not set in stone and can be modified by changes in the postnatal environment.

Several nutrient-sensing signals are involved in high-fructose and high-fat-induced hypertension, such as SIRT4, AMPKα2, PPARs, and PGC-1α. Our previous studies showed that post-weaning high-fat diet increased body weight and BP in adult male offspring, which was associated with decreased renal protein levels of phosphorylated AMPK2α and PGC-1α [20,21]. These findings are consistent with those reported in the HFR/HFA group in the current study. SIRT4, a mitochondrial sirtuin, can regulate redox metabolism, mitochondrial dynamics, and cardiovascular homeostasis [22]. AMPK is a heterotrimeric protein complex comprising a catalytic α subunit and two regulatory β and γ subunits. AMPKα2 knockout mice developed oxidative stress, endothelial dysfunction, and obesity [23]. Thus, one might expect that high-fructose and high-fat diets reduced SIRT4 and AMPKα2, via increased oxidative stress in a way that programs the development of hypertension. Our previous reports demonstrated that the PPAR signaling pathway is significantly regulated in a variety of models of programmed hypertension [24,25]. In the current study, PPARα and PPARβ isoforms were inhibited by either maternal high-fructose diet or combined post-weaning high-fructose or high-fat diets. These findings are in agreement with previous studies showing that activation of PPARα or PPARβ/δ had antihypertensive effects [24,26]. Given that PGC-1α overexpression was reported to preserve NO generation and thus lower BP [27], and that PGC-1α can promote autophagy [28], additional study is required to clarify whether targeting on PGC-1α can protect against high-fructose or high-fat intake-induced programmed hypertension. Additionally, our data demonstrated that a maternal high-fructose diet combined with a post-weaning high-fat diet, but not high-fat diet reduced *Ulk1*, *Atg5*, and *Nrf2* mRNA levels, induced a greater degree of oxidative stress and hypertension. The mechanism whereby offspring of mothers exposed to high-fructose intake are more vulnerable to develop hypertension in response to a post-weaning high-fat diet than a high-fructose diet is not clear. While mitochondria are a major source of ROS, it is presumed that selective removal of mitochondria by autophagy might be a protective effect of different post-weaning diets in offspring against oxidative stress-related hypertension.

A growing body of evidence demonstrate that an early shift in the NO-ROS balance toward reduced NO bioavailability contributes to the development of hypertension [10,17]. We found an increase in 8-OHdG staining, an oxidative stress damage marker, in the kidneys of offspring exposed to high-fructose and high-fat diets. Additionally, both diets reduced plasma l-arginine-to-ADMA ratios, an index of NO bioavailability, in offspring exposed to maternal high-fructose intake. These findings support a close link between Western diets, oxidative stress, and programmed hypertension, which concurs with previous studies from our laboratory and others [5,6,7,10,17].

Our previous report showed that maternal high-fructose diet upregulates SEH in the adult offspring kidney [5,11]. In the present study, post-weaning high-fructose and high-fat diets similarly restored SEH protein levels in adult offspring kidneys exposed to maternal high-fructose intake. As SEH inhibitors can decrease BP in various animal models of hypertension and PPARs can regulate SEH expression [14], the decreases of SEH in response to post-weaning high-fructose and high-fat diets might be a negative feedback compensatory response to the alterations of PPARs.

Our study has a few limitations. First, we did not test potential therapeutic approach. The relative importance between nutrient-sensing signals, autophagy, oxidative stress, and NO pathway in high-fructose and high-fat diets-induced programmed hypertension awaits further elucidation. Next, we did not examine other organs that help to control BP. Third, we did not conduct the HFA/ND and ND/HFA groups as we have studied their effects on BPs previously [20,21]. Furthermore, the long-term effects of combined maternal high-fructose diet and different postnatal insults on BPs deserve further evaluation. Last, emerging evidence indicates several common pathways related to high-fructose and high-fat diet contribute to developmental programming of hypertension. There is a need of further studies in common pathways, such as inflammation and the renin-angiotensin-aldosterone system to elucidate their roles on programmed hypertension.

## 5. Conclusions

In conclusion, post-weaning high-fat diet increases the vulnerability to develop programmed hypertension induced via maternal high-fructose intake in male offspring. However, there is a negligible synergy between maternal and post-weaning high-fructose diets on BPs. Some important mechanisms participating in the hypertensive effects of post-weaning high-fructose and high-fat diets on offspring kidneys exposed to maternal high-fructose diet, including increased oxidative stress, reduction of l-arginine-to-ADMA ratios, alterations of nutrient sensing signals, and reduction of autophagy. There is clearly a close link between nutrient sensing signals, oxidative stress, and SEH underlying the development of hypertension in response to high-fructose and high-fat intake. Additionally, there is a strong requirement to reconcile the interplay between prenatal and postnatal nutritional insults in the generation of hypertension, to develop a novel therapeutic approach for programmed hypertension induced by the widespread increase in dietary fructose and fat consumption in pregnant women and their children.

## Figures and Tables

**Table 1:** Quantitative real-time polymerase chain reaction primers sequences.

Gene	Forward	Reverse
*Sirt1*	5 tggagcaggttgcaggaatcca 3	5 tggcttcatgatggcaagtggc 3
*Sirt4*	5 ccctttggaccatgaaaaga 3	5 cggatgaaatcaatgtgctg 3
*Prkaa2*	5 agctcgcagtggcttatcat 3	5 ggggctgtctgctatgagag3
*Prkab2*	5 cagggccttatggtcaagaa 3	5 cagcgcatagagatggttca 3
*Prkag2*	5 gtgtgggagaagctctgagg 3	5 agaccacacccagaagatgc 3
*Ppara*	5 agaagttgcaggaggggatt 3	5 ttcttgatgacctgcacgag 3
*Pparrb*	5 gatcagcgtgcatgtgttct 3	5 cagcagtccgtctttgttga 3
*Pparg*	5 ctttatggagcctaagtttgagt 3	5 gttgtcttggatgtcctcg 3
*Ppargc1a*	5 cccattgagggctgtgatct 3	5 tcagtgaaatgccggagtca 3
*Nrf2*	5 cccattgagggctgtgatct 3	5 tcagtgaaatgccggagtca 3
*Ulk1*	5 gagtacccgcaccagaatgt 3	5 gctgtgtagggtttccgtgt 3
*Atg5*	5 ttggcctactgttcgatcttctt 3	5 ggacagtgcagaaggtcctttt 3
*Ephx2*	5 cacagcctcggctttgaga 3	5 tcacatacccatggctgacatc 3
*Rn18s*	5 gccgcggtaattccagctcca 3	5 cccgcccgctcccaagatc 3

**Table 2:** Antibodies used for Western blotting.

Antibody	Host	Source	Product Number	Dilution
pAMPKα2	Rabbit	Santa Cruz Biotechnology	SC-33524	1:1000
PPARα	Rabbit	Abcam plc.	ab8934	1:1000
PPARβ	Mouse	Santa Cruz Biotechnology	SC-74517	1:1000
PGC-1α	Rabbit	Santa Cruz Biotechnology	SC-13067	1:1000
SEH	Rabbit	Santa Cruz Biotechnology	SC-25797	1:1000

pAMPKα2: phosphorylated 5′ adenosine monophosphate-activated protein kinase 2α; PPARα: peroxisome proliferator-activated receptor α; PPARβ: peroxisome proliferator-activated receptor β; PGC-1α: PPAR-γ coactivator-1α; SHE: soluble epoxide hydrolase.

**Table 3:** Summary of weight, blood pressures, and functional parameters in male offspring exposed to maternal high-fructose intake and post-weaning high-fat diet at 12 weeks of age.

Groups	ND/ND	HFR/ND	HFR/HFR	HFR/HFA
Mortality	0%	0%	0%	0%
Body weight (g)	407 ± 8	398 ± 10	355 ± 18 ^a,b^	407 ± 13
Left kidney weight (g)	1.96 ± 0.09	1.68 ± 0.05 ^a^	2.00 ± 0.14	1.57 ± 0.1 ^a^
Left kidney weight/100 g body weight	0.38 ± 0.01	0.42 ± 0.01	0.58 ± 0.05 ^a,b^	0.39 ± 0.02 ^c^
Systolic blood pressure (mm Hg)	140 ± 2	154 ± 4 ^a^	155 ± 2 ^a^	167 ± 5 ^a,b,c^
Diastolic blood pressure (mm Hg)	82 ± 2	80 ± 2	90 ± 3 ^a,b^	96 ± 4 ^a,b^
Mean arterial pressure (mm Hg)	101 ± 1	104 ± 1	112 ± 2 ^a,b^	119 ± 4 ^a,b,c^
Creatinine (μM)	19.9 ± 0.6	16.2 ± 0.6	16.1 ± 1.7	18.3 ± 1.3

HFR/ND, maternal high-fructose intake; HFR/HFR, maternal high-fructose plus post-weaning high-fructose intake; HFR/HFA, maternal high-fructose plus post-weaning high-fat intake. *n* = 8/group. ^a^ *p* < 0.05 vs. ND/ND; ^b^ *p* < 0.05 vs. HFR/ND; ^c^ *p* < 0.05 vs. HFR/HFR.

**Table 4:** Plasma levels of l-arginine, l-citrulline, ADMA, and SDMA in male offspring exposed to maternal high fructose intake and post-weaning high-fat diet at 12 weeks of age.

Groups	ND/ND	HFR/ND	HFR/HFR	HFR/HFA
l-Arginine (µM)	288.3 ± 6.7	226.4 ± 9.1	135.1 ± 2.3 ^a,b^	150.3 ± 4.0 ^a,b^
l-Citrulline (µM)	57.2 ± 1.1	48.5 ± 1.5	43.2 ± 1.2	56.6 ± 1.6
ADMA (µM)	0.97 ± 0.02	1.06 ± 0.04	0.81 ± 0.01	0.86 ± 0.01
SDMA (µM)	0.61 ± 0.01	0.58 ± 0.01	0.5 ± 0.01	0.52 ± 0.01
l-Arginine to ADMA ratio (µM/µM)	226 ± 3	229 ± 11	167 ± 1 ^a,b^	175 ± 4 ^a,b^

HFR/ND: maternal high-fructose intake; HFR/HFR: maternal high-fructose plus post-weaning high-fructose intake; HFR/HFA: maternal high-fructose plus post-weaning high-fat intake; ADMA: asymmetric dimethylarginine; SDMA: symmetric dimethylarginine; *n* = 8/group; ^a^ *p* < 0.05 vs. ND/ND; ^b^ *p* < 0.05 vs. HFR/ND.
